# Positive Program Evaluation and Health Maintenance among Post-Metabolic and Bariatric Surgery Patients Following a 6-Week Pilot Program

**DOI:** 10.1007/s11695-023-07013-3

**Published:** 2024-01-03

**Authors:** Sydney McIntosh, Madison Hayes, Makenzie L. Barr-Porter

**Affiliations:** Department of Dietetics and Human Nutrition, University of Kentucky, Lexington, KY 40506 USA

**Keywords:** Post-bariatric surgery, Nutrition, Support, Pilot, Feasibility, Acceptability

## Abstract

**Purpose:**

Despite lifestyle changes and medication therapies, weight loss is difficult to maintain. Metabolic and bariatric surgery (MBS) is an effective route for significant weight reduction. However, post-operation there are limited opportunities to support weight loss maintenance. The following study aimed to pilot test a 6-week, 6-session nutrition and support program for post-MBS surgery patients.

**Materials and Methods:**

A 6-week post-MBS pilot nutrition and support program was developed to test feasibility and acceptability. Participants completed a baseline survey that included demographics, weight changes, success post-surgery, and self-efficacy of leading a healthy lifestyle. Weight change, percent total weight loss, self-efficacy, and program evaluation measures were assessed.

**Results:**

Participants (*n* = 18) were recruited from a local MBS clinic, predominately female (88.9%), non-Hispanic white (94.4%), received sleeve gastrectomy surgery (100%), and were 2–3 years post-operation (44.4%). Eight of the 18 participants attended the in-person sessions, serving as the intervention group. Both weight loss over and behavioral variables remained stable for both groups across the 6 weeks with no significant differences from pre to post program (*p* > 0.05). For program evaluation, intervention participants “agreed” or “strongly agreed” with 10 of 11 program satisfaction measures.

**Conclusion:**

Following the program, weight loss was maintained among both intervention and control groups. Intervention feedback indicated that the program’s approach to provide nutrition education and support was successful and acceptable. Future enhancement of the intervention should include a broader multidisciplinary approach, longer intervention period, and intentional recruitment of participants with a weight regain.

**Graphical Abstract:**

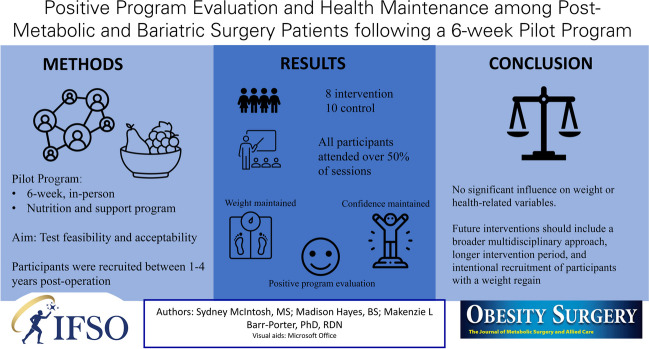

## Introduction

Obesity, as a chronic metabolic disease, can be difficult to treat due to a multitude of factors. Consistent change over an extensive period is needed to assist in reducing the burden of obesity. However, for many, lifestyle or behavior-based treatment may not achieve lasting results. For those unable to achieve lasting weight loss through their own personal mechanisms, metabolic and bariatric surgery (MBS) has been recognized as an effective and safe treatment to promote sustainable weight loss through restrictive and/or malabsorptive procedures [1–3]. Updated guidelines recommend metabolic and bariatric surgeries for patients with a body mass index (BMI) of 35 kg/m^2^ or more, regardless of obesity-related comorbidities displayed [4–6].

The most common types of metabolic and bariatric surgeries performed in America are the vertical sleeve gastrectomy (58.1% of MBS in 2021) and the Roux-en-Y gastric bypass (21.5% in 2021) [4]. Weight loss surgeries, in addition to significant body weight reduction, have been indicated to assist in the alleviation of obesity-related comorbidities such as hypertension, type 2 diabetes, heart disease, dyslipidemia, sleep apnea, nonalcoholic fatty liver disease, certain cancers, and many other unfavorable afflictions [7–9].

Though MBS can assist in significant health improvements, weight loss surgery requires significant lifestyle and behavior changes following the procedure to improve rates of success, sustain resolution of comorbidities, and long-term weight loss [10]. Due to the nature of post-operative metabolic and bariatric surgery care and maintenance, the National Institutes of Health (NIH), American College of Surgeons (ACS), and American Society for Metabolic and Bariatric Surgery (ASMBS) have developed guidelines for clinics and patients, including advising patients to seek metabolic and bariatric surgery care at a clinic with a multidisciplinary team [4–6]. The specific multidisciplinary team must include a board-certified surgeon, a registered dietitian, an exercise specialist, and a mental health professional all specialized in metabolic and bariatric surgery [4–6].

Specifically, registered dietitians play an important role in influencing favorable health outcomes for the patient both before, and following, MBS [11, 12]. Throughout MBS cases, a registered dietitian may assist in eligibility screening of patients to receive MBS, evaluating and correcting nutrient deficiencies through medical nutrition therapy, assessing the patient’s readiness to change, educating the patient on proper physical activity and eating techniques, and making appropriate dietary recommendations including supplementation and diet progression post-operation [11, 13, 14]. Post-operation, a patient may follow up with a registered dietitian for assistance in sustaining diet and lifestyle [13]. Patients who follow up with a registered dietitian after surgery tend to have fewer hospital readmissions related to nutrient deficiencies, greater resolution of comorbidities, and greater weight loss [13, 15]. However, although recommended by previous research and MBS-related organizations, there are no requirements to meet with a registered dietitian during the post-operation period [16].

Of the patients who undergo metabolic and bariatric surgery, 20–30% may have inadequate weight loss [17]. This inadequate weight loss, loosely defined as losing less than 20% of their total weight, can be influenced by diet and lifestyle nonadherence after surgery and could be a consequence of a lack of social and clinical support [13, 15, 18]. Support programming after surgery, though limited across the board, has proven to be beneficial to post-metabolic and bariatric surgery patients to encourage optimum success in achieving sustainable weight loss and adhering to dietary and lifestyle post-operation [13, 15]. However, curriculum and guidelines for these post-operative support programs are limited and beg further research.

The current study aimed to pilot-test a nutrition and support program for post-metabolic and bariatric surgery patients. The specific aim of this study was to determine if a six-session nutrition-based program created for post-operative bariatric surgery patients is acceptable for promoting nutritional and social support and maintaining weight.

## Methods

A six-session pilot program was designed to engage post-operative MBS patients that received surgery from a local Kentucky clinic between 1 and 4 years prior. Curricula were developed to provide post-operative nutrition information and social support. Data were collected from participants at baseline, weekly at the intervention sessions, and post-program. The study was approved by the University of Kentucky’s Institutional Review Board (#77600). Informed consent was obtained from all individual participants included in the study.

### Recruitment and Procedures

The six-session pilot program was conducted in person at a mid-size bariatric surgery clinic where patients previously received their care. Post-operative patient participants were recruited through the clinic’s listserv by a registered dietitian on staff. The participants who met the eligibility criteria (18 years of age or older, had bariatric surgery within the last 1–4 years, had the sleeve gastrectomy or Roux-en-Y gastric bypass as their initial MBS procedure, can read, speak, and understand the English language, and were not currently pregnant) were invited to complete the baseline survey. After the completion of the baseline survey, the participants were invited to attend a six-session pilot program. The pilot program curriculum was established in collaboration with a registered dietitian with expertise in bariatric surgery, a nutrition graduate student, and a nutrition undergraduate researcher utilizing evidence-based resources. The program consisted of six sessions related to nutrition and support (Table 1). Each session of in-person group sessions included a short PowerPoint presentation to assist with information dissemination, followed by session activities and group discussions.

**Table 1:** Six sessions related to nutrition and support

Session	Topic	Content overview
1	Introduction	Overview of the program, research, and gathered participant input on future topics and information
2	Post-operative nutrition guidelines	Nutrition recommendations, example meals and snacks, helpful tips and suggestions for shopping, budgeting, and choosing foods
3	Micro and macronutrient recommendations for post-bariatric surgery	Importance of nutrients, where to find nutrients, importance of multivitamin compliance, and high micronutrient food examples
4	Activity and mindfulness	Group walks around the facility, chair yoga, and deep breathing. Engagement in a mindful eating exercise, discussed mindful self-care activities such as journaling, meditation, and yoga
5	Individual and social support	Invitation to bring a support person(s) to the session as well as engage in intersession support discussions
6	Sustainability of behavioral change	Maintaining motivation, working toward goals, sustaining behavior change long term

The study in-person group program was held once weekly, across six sessions, for approximately 1 h in the evenings at the surgery clinic. The program was held in a conference room with space for interaction along with television access to ensure sharing of educational materials. Printed materials (flyers, pamphlets, PowerPoint slides) and activities (take-home activities, journaling, physical exercise) were shared weekly related to the topic.

### Measures

Participants completed a baseline survey online through REDCap that included demographic data (age, biological sex, race and ethnicity, surgery type, and surgery date), when weight regain, insurance type, comorbidities (pre-and post-surgery, and employment), weight-specific demographic data (weight before surgery, current weight, and weight regain), perceptions of personal surgery success in weight loss, diet, and physical activity (ex. “I have had success with choosing healthy foods after surgery”), and self-efficacy of healthy lifestyle adherence (ex. “I am confident I am able to be a healthy version of myself”). Following the baseline survey, participants were invited to enroll in the six-session in-person program. Data for intervention participants were only included in pre- and post-measures for those who attended at least 50% of the in-person sessions (3 of 6 sessions).

Percent total weight loss (%TWL) and excess weight loss (%EWL) are both accepted measures of weight loss success. %TWL was established through ((starting weight − current weight/starting weight) multiplied by 100) [19]. %EWL was calculated as (weight before surgery − weight prior to intervention)/(starting weight) × 100 [19]. %EWL and %TWL were calculated using self-reported data from the baseline survey.

Weight was taken each session via Tanita electrical impedance scale. Weight from physical measurements was used to assess the reliability of self-reported weight.

To understand satisfaction with the approach used in this program, individuals participating in the in-person group program were asked questions regarding their feedback on program satisfaction and acceptability. To avoid excessively neutral response patterns, each item was scored on a 6-point Likert scale (1 = strongly disagree, 6 = strongly agree) with no neutral mid-point [20]. Examples of questions that were asked regarding program satisfaction and acceptability include “I am satisfied with the program approach used to provide nutrition and support for post-bariatric surgery,” “The in-person program was useful for gaining nutrition knowledge,” and “the in-person program provided a sense of social support.”

### Statistical Analysis

Distributions were run on all continuous variables for normality using Shapiro–Wilk Goodness of Fit test. Descriptive statistics, including frequencies and measures of central tendency (means and standard deviation), are shown for the entirety of the cohort of post-operative bariatric surgery patients who completed the baseline survey. All descriptive categorical data was analyzed through Pearson chi square test. Continuous demographic variables (age and total conditions before and after the procedure) are continuous nonnormal variables and analyzed using Mann Whitney *U*.

Weight-specific baseline survey data (weight before surgery (pounds), current weight (pounds), and weight regain (pounds)) were compared from pre- to post-program between the control participants and the intervention participants who attended at least 50% of the sessions using an independent *t*-test.

Spearman’s rho correlations were used to assess agreement between pre- and post-Tanita weight and self-reported weight from survey responses. Due to the strong correlation between the two, self-reported measures were used for further analyses (pre *ρ* = 0.98, *p* < 0.01; post *ρ* = 1.00, *p* < 0.01).

Percent total weight loss and percent excess weight loss from pre- to post-program were analyzed using an independent *t*-test.

Self-efficacy of healthy lifestyle adherence and perceptions of personal surgery success were shown as continuous variables of change from pre to post of response by Likert items used on a continuous scale from 1 to 7 (strongly agree to strongly disagree). Questions regarding perceptions of success and self-efficacy were analyzed through a Wilcoxon rank sum test to determine changes from pre-to-post-intervention.

Program evaluation survey questions were asked on 6-point Likert items (1 = strongly disagree to 6 = strongly agree) to avoid neutral response. Data was shown through means and standard deviations of responses.

## Results

Twenty-three participants consented into the study. Ten participants self-selected to not participate in the in-person portion and are further used as the control group. Thirteen of the 23 participants self-selected to participate in the in-person support and nutrition-based pilot intervention over six sessions. Eight of the 13 attended at least 50% of the intervention, thus serving as the “intervention group” and were used for analysis. A final sample of 8 intervention, and 10 control were used. In addition to two baseline surveys, the in-person participants completed a post-program success and acceptability survey and participated in session data collection.

Demographics of the participants are displayed in Table 2. There were no statistically significant differences between the participants’ characteristics found between the intervention and control groups.

**Table 2:** Descriptive characteristics of intervention and control participants (*n* = 18)

Variable and category	Total (*n* = 18)	Intervention (*n* = 8)	Control (*n* = 10)	*p* value
Age (years) mean ± SD	44.67 ± 10.47	50.13 ± 11.04	40.30 ± 8.04	0.0558
Sex (%)				0.8668
Female	16 (88.89)	7 (87.50)	9 (90.00)	
Male	2 (11.11)	1 (12.50)	1 (10.00)	
Race (%)				0.3574
African American or Black	1 (5.56)	0	1 (10.00)	
Caucasian	17 (94.44)	8 (100)	9 (90.00)	
Ethnicity (%)				0.2451
Hispanic or Latino	1 (5.56)	1 (12.50)	0	
Not Hispanic or Latino	16 (88.89)	6 (75.00)	10 (100)	
Unsure	1 (5.56)	1 (12.50)	0	
Surgery type (%)				
Sleeve gastrectomy	18 (100)	8 (100)	10 (100)	
Surgery date (%)				0.7985
Between 1 and 2 years	6 (33.33)	2 (25.00)	4 (40.00)	
Between 2 and 3 years	8 (44.44)	4 (50.00)	4 (40.00)	
Between 3 and 4 years	4 (22.22)	2 (25.00)	2 (20.00)	
When weight regain began				0.3211
N/A	3 (16.67)	0	3 (30.00)	
9 months	1 (5.56)	1 (12.50)	0	
12 months	4 (22.22)	2 (25.00)	2 (20.00)	
18 months	6 (33.33)	2 (25.00)	4 (40.00)	
2 years	1 (5.56)	1 (12.50)	0	
2.5 years	3 (16.67)	2 (25.00)	1 (10.00)	
Insurance type				0.1781
Blue Cross Blue Shield	8 (47.06)	4 (50.00)	4 (44.44)	
Medicaid	3 (17.65)	1 (12.50)	2 (22.22)	
WellCare	3 (17.65)	0	3 (3.33)	
Other	2 (11.76)	2 (25.00)	0	
None	1 (5.88)	1 (12.50)	0	
Conditions				
Total conditions before surgery (frequency)	2.94 ± 2.15	3.75 ± 1.16	2.30 ± 2.58	0.1227
Total conditions after surgery (frequency)	2.16 ± 2.38	2.13 ± 1.46	2.20 ± 3.01	0.4637
Employment				
Full time	12 (60.00)	4 (20.00)	8 (40.00)	0.1797
Part time	0	0	0	0.0000
Unemployed	1 (5.00)	1 (5.00)	0	0.2500
Disabled	2 (10.00	1 (5.00)	1 (5.00)	0.8668
Retired	2 (10.00)	2 (10.00)	0	0.0935
Self-employed	0	0	0	0.00
Homemaker	2 (10.00)	1 (5.00)	1 (5.00)	0.8668
Student	1 (5.00)	1 (5.00)	0	0.2500

The Pearson chi square test was used to analyze categorical demographic variables. Mann Whitney *U* was used to analyze continuous demographic variables

An independent *t*-test was used to determine the difference in weight variables between the intervention and control participants which is shown on Table 3. Due to small sample size, weight was assessed by sex to understand any sex-related differences. Weight was found to be significantly higher in males than females (*p* value = 0.0046). On average, the intervention participants’ body weight was 290.13 lbs (SD 64.90) before surgery. The control group’s average body weight was 313.00 lbs (SD 79.49) before surgery, not significantly different from intervention (*p* = 0.53). The self-reported current weight of the intervention participants at baseline was 209.38 lbs (SD 42.51) and the control reported currently weighing 210.00 lbs (SD 70.38) (*p* = 0.79). Since receiving MBS, the intervention group self-reported regained 16.25 lbs (SD 9.48), and the control participants regained 13.00 lbs (SD 15.84) (*p* = 0.26).

**Table 3:** Weight-specific descriptive characteristics of intervention and control participants at baseline

Variable	Intervention (*n* = 8)	Control (*n* = 10)	*p* value
	Mean ± SD	Mean ± SD	
Weight before surgery (lbs)	290.13 ± 64.90	313.00 ± 79.49	0.5335
Current weight (lbs)	209.38 ± 42.51	210.00 ± 70.38	0.7898
Weight regained after surgery (lbs)	16.25 ± 9.48	13.00 ± 15.84	0.2645

Independent *t*-test test was used to assess differences by intervention group

Independent *t*-test was used to analyze differences in %TWL and %EWL by time and by intervention group due to the nonparametric characteristics of the data. As shown in Table 4, the differences in %EWL and %TWL change from pre-to-post between the intervention and control participants were not statistically significant. Based on the EWL standards of weight loss success (%EWL ≥ 50%) and the %TWL standards (%TWL ≥ 20%), both the intervention and control participants were successful before and after the program [21]. %EWL and %TWL remained stable across the six sessions. Though control participants had a greater %TWL than the intervention group to begin with, there was not a significant difference at baseline between the groups (*p* = 0.40).

**Table 4:** Success and changes in weight from pre to post-intervention

Variable	Pre-intervention	Post-intervention	*p* value by time
	Mean ± SD	Mean ± SD	
%EWL change			
Intervention	62.07 ± 16.53	64.76 ± 15.32	0.7413
Control	67.74 ± 28.69	69.23 ± 28.61	0.9090
*p value by group*	*0.6074*	*0.6781*	
%TWL change			
Intervention	27.35 ± 6.12	28.50 ± 4.08	0.6662
Control	33.28 ± 12.16	34.06 ± 12.18	0.8880
*p value by group*	*0.2004*	*0.2027*	

Independent *t*-test was used to assess differences by intervention group and by time

*%EWL* percent excess weight loss, *%TWL* percent total weight loss

Perceptions of success and self-efficacy in leading a healthy lifestyle were assessed by the Wilcoxon rank sum test (Table 5**)** by group and by time. On average, perceptions of success and confidence to pursue bariatric-specific healthy lifestyle procedures were maintained throughout the program among both the intervention and control participants. Through analysis, it was found that at baseline, the control participants reported being statistically significantly more confident to be the healthiest versions of themselves than the intervention participants.

**Table 5:** Self-efficacy changes from pre-intervention to post-intervention by control

Variable (range of answers)	Pre	Post	*p* value
	(Mean ± SD)	(Mean ± SD)	
Success with weight loss after surgery			
Intervention (1–3)	2.25 ± 0.89	1.75 ± 0.71	0.2207
Control (1–3)	1.70 ± 0.82	1.50 ± 0.71	0.5840
Success with physical activity after surgery			
Intervention (1–5)	2.75 ± 1.58	2.38 ± 1.41	0.6650
Control (1–4)	2.50 ± 1.43	2.70 ± 1.49	0.6941
Success with choosing healthy foods after surgery			
Intervention (1–5)	2.75 ± 1.28	3.00 ± 1.41	0.7037
Control (1–4)	2.30 ± 0.67	2.10 ± 0.88	0.4028
Confidence in the ability to eat healthily			
Intervention (1–7)	3.13 ± 1.55	3.50 ± 2.07	0.7888
Control (1–5)	2.50 ± 1.18	2.10 ± 0.74	0.4458
Confidence in the ability to exercise			
Intervention (1–6)	3.25 ± 1.67	2.75 ± 1.49	0.5898
Control (1–7)	2.80 ± 1.69	2.40 ± 1.17	0.5508
Confidence to be healthy			
Intervention (1–6)	3.88 ± 1.73^	2.63 ± 1.41	0.1347
Control (1–4)	2.10 ± 0.99^	2.10 ± 0.74	0.8719

The Wilcoxson rank sum test was used to assess perceived success and confidence by group and by time. Likert item questions from 1 = strongly agree to 7 = strongly disagree

^*^p < 0.05

^Significant difference between groups at baseline

Intervention participants (*n* = 8) completed a post-program survey on the acceptability and success of the intervention. Results from the survey are shown in Table 6. Ten of the 11 evaluation questions inspired predominately strongly agree and agree on responses regarding the success and acceptability of the program.

**Table 6:** Post-program evaluation

Question number	Question	Intervention (*n* = 7)
		(Mean ± SD)
1	I am satisfied with the program approach used to provide nutrition and support for post-bariatric surgery	5.86 ± 0.38
2	The program length was reasonable (6 sessions, 1 h each)	5.71 ± 0.49
3	The in-person program was useful for gaining nutrition knowledge	5.71 ± 0.49
4	The in-person program provided a sense of social support	6.00 ± 0.00
5	The in-person program was helpful for accountability after bariatric surgery	5.71 ± 0.76
6	The in-person program was useful for recommendations on overall healthy living	5.71 ± 0.49
7	This program improved my confidence in living a healthy lifestyle	5.29 ± 0.76
8	I have changed my diet habits throughout the program	5.00 ± 1.15
9	I have not made any changes yet, but plan to make dietary changes following this program	3.71 ± 1.80
10	I plan to use information from this program in my lifestyle moving forward	5.43 ± 0.79
11	I would recommend this in-person program for post-bariatric surgery individuals	5.71 ± 0.49

Post-program questions were asked on a Likert scale item of (1 = strongly disagree, 6 = strongly agree)

## Discussion

Over the six sessions, participants who engaged in the in-person program maintained body weight, perceived confidence, and self-efficacy to lead a healthy post-metabolic and bariatric surgery lifestyle. Participant feedback derived from the post-program evaluation survey regarding the success of the post-MBS program supported the specific aim that a six-session post-MBS program was acceptable in providing nutrition education and support. Although data collected failed to see significant changes from pre-to-post-program, this intervention contributes to post-metabolic and bariatric surgery programs and warrants future investigation into long-term support for these unique patients. Findings from this study can be used as a tool in creating future programs to offer support and nutrition education for post-metabolic and bariatric surgery patients.

Although not significant, weight maintenance was observed in both the control and intervention participants. Generally, 20–30% of post-MBS patients struggle with success in weight loss following surgery [13, 17, 22]. Though the measure of success in weight loss is controversial, and not yet standardized, percent total weight loss (%TWL) is a commonly utilized approach [11, 18, 23]. Recent research has established success in weight loss post-MBS as a TWL ≥ 20% [18]. According to the success in weight loss standards, both the intervention and control were successful in weight loss both prior and following the intervention. Based on the findings of this research, future studies should include a standard of success in weight loss considering inclusion criteria by recruiting patients who are below that threshold and may benefit most from these types of programs [24, 25]. To continue, another adaptation of this study that could promote weight loss is changing the time period after surgery. Due to recruitment difficulties, the study was opened to participants who received surgery between 1 and 4 years ago. Consideration in future studies should be taken for utilizing a wide-range of post-operation years. Additionally, in consideration of year grouping of participants, larger sample sizes in each group would be warranted. In addition, recent research suggests that post-MBS nutrition-based programs could be efficient in promoting weight loss when conducted directly after surgery and continued for a longer length of time [26, 27].

When aiming to understand an individual’s behaviors, dietary change, and motivation, self-efficacy is a common measure to assess. Bandura defined self-efficacy as a person’s perceived ability to perform a certain behavior [28, 29]. The use of behavior change theories as a framework for lifestyle interventions is supported by current findings [30, 31]. The current study utilized self-efficacy when establishing the curriculum for the study; however, when analyzing the results regarding self-efficacy and behavior change, there was no significant evidence proving the intervention promoted either variable. This lack of confidence change following the intervention could be due to the short period of time or an increase in raised awareness of current behaviors. There is potential that participants self-perceive their behaviors as generally healthy before participating in a nutrition and support program. Following nutrition-related reminders and peer sharing, intervention participants may feel like they are not following the recommended guidelines as well as they originally thought. Adaptations to the post-program survey to include consciousness-raising questions would be a beneficial enhancement to control for these potential components.

Though this intervention was aimed to target nutrition and general support, the inclusion of a multidisciplinary team such as mental health professionals, in addition to a registered dietitian, can assist in the promotion of behavior change [32]. A comparable intervention found significant results regarding eating self-efficacy after a 10-week intervention that included both a registered dietitian and a social worker [32]. Longer intervention periods (3–6 months) could have stronger implications for health and behavior-related outcome. However, participant burden does need to be considered in the length of time an intervention is held. Similar interventions have found significant results regarding improved changes in eating self-efficacy and weight loss following a 10-week intervention [32, 33], though ongoing support has the potential to provide the most desirable results in terms of weight loss and behavior change [23, 34].

The feedback collected from the intervention participants supported the program’s aim that a six-session pilot support and nutrition-based program for post-MBS patients was acceptable for promoting nutrition information and support. Comparable pilot studies found similar results in terms of acceptability, feasibility, and demonstrating the need for more research to support this population [35, 36]. Ten of the 11 items of the post-program success and acceptability survey inspired either strongly agree or agree on ratings. Notably, one evaluation statement, “The in-person program provided a sense of social support,” received “strongly agree” responses from all the participants. A comparable intervention found that there is a positive sense and need for social support among this population [37, 38]. To continue, the question “The in-person program was useful for gaining nutrition knowledge” also received positive feedback. This question is important to note for future research because the literature supports that acquiring nutrition education can increase healthy lifestyle habits and healthy food consumption [39]. Research suggests that food choices can be influenced by a variety of factors, but nutrition and health education programs positively create awareness and motivation to undergo healthier lifestyles [39]. Though MBS patients typically undergo several sessions of nutrition education before surgery, there is a lack of nutrition support post-MBS due to clinic burden and a diminished “requirement” by insurance companies or clinics for participants to maintain attendance at follow-up appointments. Continuation of support through various modalities has the ability to reach more patients in an efficient manner.

Finally, of note, one program evaluation question, “I have not made any changes yet, but plan to make dietary changes following this program,” received the lowest among the scores for the items. Researchers believe that the wording of this statement may have led to confusion by participants in which the statement could have been perceived as the participant may have already made changes throughout the program, so they would rate this toward “disagree.” It is recommended to reword the statement to either clarify or purposefully keep it negatively worded to ensure participants are accurately reading and answering statements.

Strengths of this six-session pilot study include having a registered dietitian specializing in metabolic and bariatric surgery on the research team and conducting the in-person group session at the site in which treatment was received. The utilization of multiple modes of education and learning in the program seemed to enhance peer engagement and interaction with the material. The use of PowerPoint, printed handouts, and hands-on exercise and activities allowed for further discussion among the group. Likewise, it is clear that the program was viewed in a positive light by participants and was evaluated favorably in the sense of modality, duration, ability to support them post-operatively, and providing useful information. Lifestyle behaviors and weight were maintained across the intervention as well, indicating that participants were able to attenuate any adverse health outcomes across the timeframe.

### Limitations

Due to the pilot nature of this study, limitations were identified in the approach and described throughout the discussion. The sample population was predominately female, which previous research has found that post-MBS female patients are more likely to adhere to post-operative guidelines [32, 40, 41]. Likewise, individuals who self-selected to participate in this program were likely interested in improving their outcomes. The population recruited was a convenience sample and was deemed “successful” in the outline of %TWL of 20%. Consideration should be taken when recruiting a sample for a larger intervention. Recruitment of participants with a specific range of weight regain (i.e., 5–10% of weight regained since nadir weight post-operation) could identify if the program would be effective in weight reduction rather than just weight maintenance. Collection of additional health parameters (A1c, lipid profile, etc.) could also provide more information on the sample and potential metabolic changes as it relates to program efficacy rather than relying solely on weight as a success parameter.

This pilot study recruited a small sample size and data used in the analyses were taken from intervention participants who completed at least 50% of the program sessions, which compares to a similar pilot study [32]. Poor follow-up attendance in clinic appointments and post-MBS programs has been a historically discussed topic. Understanding the factors that influence attendance to decrease could provide further understanding of the approaches needing to be explored in research programs to entice participants to continue with post-MBS programs [32, 42]. Additionally, though contradictory, the length of the program was shorter than other post-MBS programs. This length of program was consciously chosen to reduce attrition by reducing the time commitment and ensure timing in the calendar year was not impacted by significant holidays. However, a longer intervention period of 3 to 6 months could be beneficial to allow participants to further engage with material, feel more comfortable with their peer group, have stronger changes to health and lifestyle factors, and improve their self-efficacy for health and well-being over holidays and in various spaces of the year.

Due to the pilot nature of the project, a program evaluation tool was tailored and developed to fit the program and population. The utilization of a valid and reliable measure should be employed in future studies or the reliability testing of the current measure could be tested.

Finally, it has been shown that multidisciplinary team members can enhance the care, support, and positive outcomes for various health conditions and concepts within health-related interventions. The current intervention focused on a nutrition and peer support approach to the curriculum and thus lacked additional multidisciplinary support. Throughout conversations during the program, various aspects of food and mental health came to light which could have benefited from the addition of mental health professionals. Further adaptation of this program would benefit from additional sessions with multidisciplinary team members to inspire deeper conversations and a more holistic approach. Particularly, among areas of diverse population spread, the inclusion of a bilingual member of the multidisciplinary team would be beneficial in increasing reach and inclusivity in the program.

## Conclusion

In this pilot study, there were no significant changes in weight, behavior, or self-efficacy, though there was maintenance across the 6 weeks. Among program evaluation, participant feedback strongly agreed that this approach was acceptable and useful. Future expansions to this research should consider lengthier durations (i.e., 3–6 months) to be responsive to the consistent need for social and clinical support following this major lifestyle surgery. In addition, creating a lifestyle program for patients within a year post-operation could inspire a routine and increase self-efficacy during a critical period of weight change. Expanding modalities of programs, including one-on-one, virtual, and/or web applications, could enhance reach, particularly to those geographically isolated. In addition, the inclusion of a multidisciplinary team throughout the program can influence a well-rounded support experience. Barriers seen among post-MBS can go beyond the scope of a registered dietitian, and expanding the team approach could be the best response to increasing clinical support for these post-MBS patients. Lastly, future research could consider collecting more data in the baseline survey including A1c, lipid markers, and other health parameters. Overall, this intervention was successful in providing support and nutrition education based on participant feedback. This study serves as a significant contribution to post-MBS programming and the need for the continuation in this area of research.

## Data Availability

All data for this study are presented in this artcile. Data are available upon reasonable request.
